# Quantifying the honey bee dance floor: A data-driven method for defining and comparing waggle dance regions

**DOI:** 10.1371/journal.pone.0341456

**Published:** 2026-02-18

**Authors:** Byron N. Van Nest, Ashley E. Wagner, Michele L. Joyner, Edith Seier, Darrell Moore

**Affiliations:** 1 Department of Biological Sciences, University of Manitoba, Winnipeg, Manitoba, Canada; 2 Department of Biological Sciences, East Tennessee State University, Johnson City, Tennessee, United States of America; 3 Department of Mathematics and Statistics, East Tennessee State University, Johnson City, Tennessee, United States of America; University of Alberta, CANADA

## Abstract

Honey bee (*Apis mellifera*) foragers perform waggle dances inside the hive to communicate the location of profitable foraging sites to nestmates. These recruitment dances occur within a specific region of the comb, known as the dance floor, but its location and structure have historically been described only qualitatively. Here we introduce a data-driven geometric method to define and quantify the dance floor from waggle-dance coordinates. The approach combines convex hulls and confidence ellipses to produce a closed region representing the area of highest dance density and yields interpretable spatial metrics including centroid location, area, perimeter, major and minor axes, and orientation. To demonstrate the method’s performance, we applied it to 155 observations of eight colonies in observation hives differing in size and date. Using complementary univariate and multivariate analyses, the framework consistently captured approximately 91% of dances, matching historical estimates based on entrance distance, and detected systematic differences among observations associated with hive size, day, and time (e.g., size-dependent shifts in centroid position and width, and time-of-day effects on orientation), illustrating its sensitivity to experimental and temporal context. This work provides an explicit quantitative definition of the honey bee dance floor and a reproducible analytical framework for comparing spatial recruitment patterns across colonies, environments, and future experimental designs.

## Introduction

One of the most intriguing forms of animal communication is the waggle dance of honey bees (*Apis mellifera*). Successful foragers advertise to their nestmates a mathematical vector (direction and distance) to a valuable resource via a series of complex manoeuvres performed on the comb inside the dark hive [1]. These recruitment dances do not occur randomly throughout the nest but are typically concentrated in a distinct area known as the dance floor. The spatial localization of this behaviour likely enhances communication efficiency by focusing both unemployed foragers and experienced reticent foragers in the same physical region where active recruits and successful scouts perform their dances [2–4]. Yet, despite decades of research, the factors shaping the location and structure of the dance floor remain poorly understood.

In both natural and managed nests, waggle dances tend to occur near the entrance on free-hanging, empty comb, which may improve vibrational transmission [5–7]. However, free-hanging, empty comb is not required for waggle dance performance; dancing also occurs in commercial and observation hives on combs fully integrated into wooden frames, including brood comb [8–11]. Common to virtually all studies performed to date (in natural, commercial, and observation hives), the dance floor is simply described as being near the hive entrance [1,8–10,12–14]. For instance, Seeley and Towne [15] noted that 94% of dances in a 2-frame observation hive were performed within 24 cm of the hive entrance, with the highest density of dances between 4 and 18 cm from the entrance. However, additional factors have been proposed, including scent-marking by early returning foragers [16], the release of chemical cues that stimulate recruitment [17,18], and preferences for particular substrate types such as brood comb [6,10,19].

Despite this interest, the dance floor has rarely been defined quantitatively. Most studies describe it only qualitatively (e.g., “near the entrance”), limiting the ability to compare spatial recruitment behaviour across colonies, contexts, or experimental conditions. One recent approach by Wario et al. [20] used principal component analysis to summarize dance orientation, but their method does not yield a bounded spatial region or metrics that can be compared systematically across observations. In contrast, our geometric framework generates an explicit, data-driven boundary that can be directly compared across contexts, allowing direct tests of how dance-floor location and shape vary with factors such as comb structure or colony state. The method combines two geometric tools: a convex hull, which traces the outer boundary of all dance points, and a confidence ellipse, which summarizes their statistical spread. Together, these yield a closed region encompassing the core of dance activity and provide biologically interpretable metrics such as area, centroid location, orientation, and spread. To demonstrate the utility of this method, we apply it to waggle dance data from eight honey bee colonies housed in glass-walled observation hives of two sizes. We use this framework to ask whether dance floor structure (i) varies throughout the day, (ii) changes from day to day, (iii) differs among colonies, (iv) shifts over the course of the season, or (v) depends on the size of the hive. By addressing these questions with a standardized approach, we aim to clarify how contextual and colony-level factors influence spatial recruitment behaviour and to offer a method that can be adapted for other species and systems in which communication is spatially organized.

## Materials and methods

### Animals, experimental setup, and data collection

Western honey bee (*Apis mellifera* L.) colonies were housed in 8-frame (100 × 100-cm) or 4-frame (100 × 50-cm) glass-walled observation hives (Table 1) in a temperate, mid-elevation region characterized by mixed forest and seasonal floral resources. The 8-frame hives were housed in a wooden shed in an overgrown field of wildflowers, berries, and clusters of both coniferous and deciduous trees. Natural light was blocked, and overhead fluorescent lamps covered by white cloth provided dim, diffuse lighting. The 4-frame hives were housed in a small light- and temperature-controlled laboratory on a town-centre academic campus with managed lawns, flower gardens, and trees. Each colony was used once in a single trial lasting between 3 and 5 days. Hive entrances were modified so that incoming bees had access to only one side of the frame nearest the entrance, thereby restricting all waggle dances to a single comb surface. A 5 × 5-cm grid was drawn on the glass sides of the hives to assist observers in estimating dance locations. Across the eight trials, a total of 7,444 dances were analyzed (Table 1). No licenses or permissions were required to carry out this work.

**Table 1:** Details of each colony.

Colony	Hive size (frames)^*a*^	Hive location	Start date	Day-of- year^*b*^	Days of observation	Observation period	Median sunrise^*c*^	Start time^*d*^	Total dances observed
1	8	shed	6 Jun 2012	157	3	0800–2030	0611	1.82	1,693
2^*e*^	8	shed	26 Jun 2012	177	3	0800–2030	0614	1.77	1,415
3	8	shed	24 Jul 2012	205	3	0800–2030	0631	1.48	846
4	8	shed	30 Jul 2012	211	3	0800–2030	0635	1.42	493
5	4	lab	29 Jun 2013	179	3	0730–2000	0615	1.25	651
6	4	lab	8 Jul 2013	188	4	0730–2000	0619	1.18	935
7^*f*^	4	lab	18 Jul 2013	198	5	0730–2000	0627	1.05	775
8	4	lab	5 Aug 2013	216	3	0730–2000	0640	0.83	636

One trial was conducted on each colony. All colonies were unrelated and queenright except as noted.

^a^Observation hives were 2 vertical columns of either 2 or 4 frames each.

^b^Number of days from January 1^st^ for Day 1 of each trial. Subsequent days-of-year were incremented as appropriate for each day of a trial.

^c^The median sunrise across days of each trial. Variation within a trial never exceeded 3 min.

^d^Number of hours since median sunrise (beginning of observation period minus sunrise) for the first observation each day. Each subsequent start time for an observation was 2 h later.

^e^Colony 2 was queenless.

^f^Colony 7 had two queens, one supersedure queen and one derived from Colony 2.

During each observation period, we recorded the locations of all waggle dancing bees by visually scanning across the grid rows in a horizontal zigzag descending fashion, beginning with the upper quadrat farthest from the hive entrance, and ending at the hive entrance. Each time a waggle dance was observed, we noted the location of the waggle run’s onset on a printed map. Because dance locations were sometimes marked beside grid lines on printed maps rather than directly on the grid lines themselves, this procedure produced a minor measurement artifact characterized by an apparent underrepresentation of dances along grid lines (see Figs 4 and 5). The same scan-sampling and marking procedure was used consistently across all colonies, observation periods, and hive sizes. This scan sampling procedure was repeated approximately 20 times per 30-minute observation period. Observations were made every 2 h starting at 0800 on Colonies 1–4 and at 0730 on Colonies 5–8. We digitized the recorded maps and calculated the horizontal and vertical distance of each dance from the hive entrance. Note that bees were not individually marked, so we were unable to account for potential repeated measures. Additionally, because hive size, time of day, and experimental setting were inherently visible during data collection, it was not possible to record data blind to treatment.

**Fig 1:**
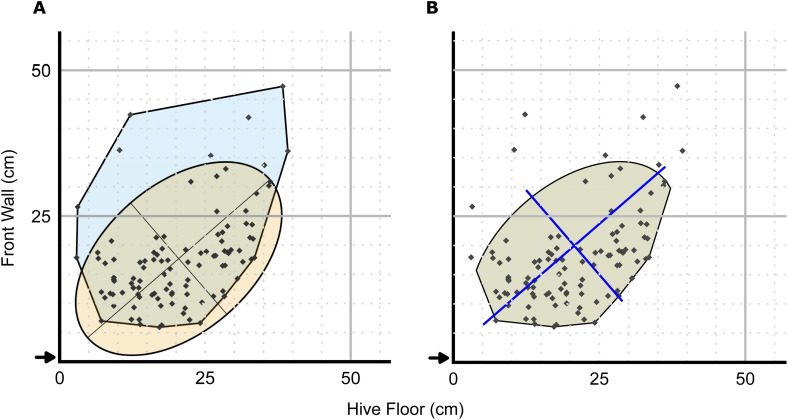
Defining the dance floor for Colony 1, Day 1, 1400 to 1430. (A) The blue+green region is the convex hull of all dances observed during the 30-minute observation. The yellow+green region is the 2-SD confidence ellipse. The major and minor axes of the ellipse are shown in black. (B) The intersection of the convex hull and the confidence ellipse from (A). The blue lines are the major and minor axes of the confidence ellipse recentred at the centroid of the dance floor. In this midday example, the dance floor captured 96 of the total 105 observed dances (91.4%), *Area* = 648.4 cm^2^, *Perimeter* = 96.3 cm, the dance floor centroid was at *X* = 20.6 cm and *Y* = 19.8 cm, *Length* (major axis) = 41.4 cm, *Width* (minor axis) = 25.3 cm, and *Angle* = 40.9° counterclockwise from horizontal. The black arrows indicate the location of the hive entrance. Individual dance locations are indicated by black dots. This example is representative of typical midday conditions, when dance activity was highest; most other observation periods exhibited similar spatial structure, with reduced definition only during early-morning or evening observation periods when dance numbers were low.

**Fig 2:**
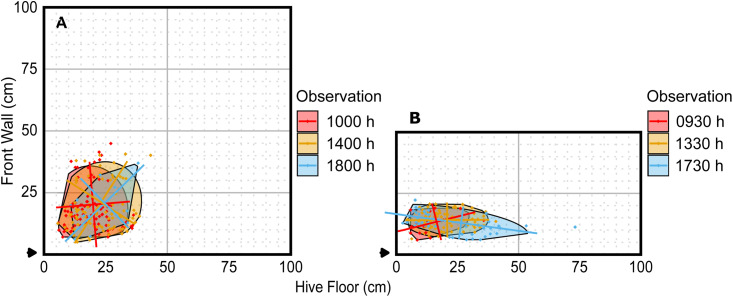
Dance floors from three observation periods from Day 1 of Colonies 2 and 5. Late morning, midday, and early evening observations are shown for 8-frame (A) and 4-frame (B) observation hives. All dance floor metrics are listed in supplemental S1 Table. Note that the angle of the major axis varies more in the 8-frame hive than in the 4-frame hive.

**Fig 3:**
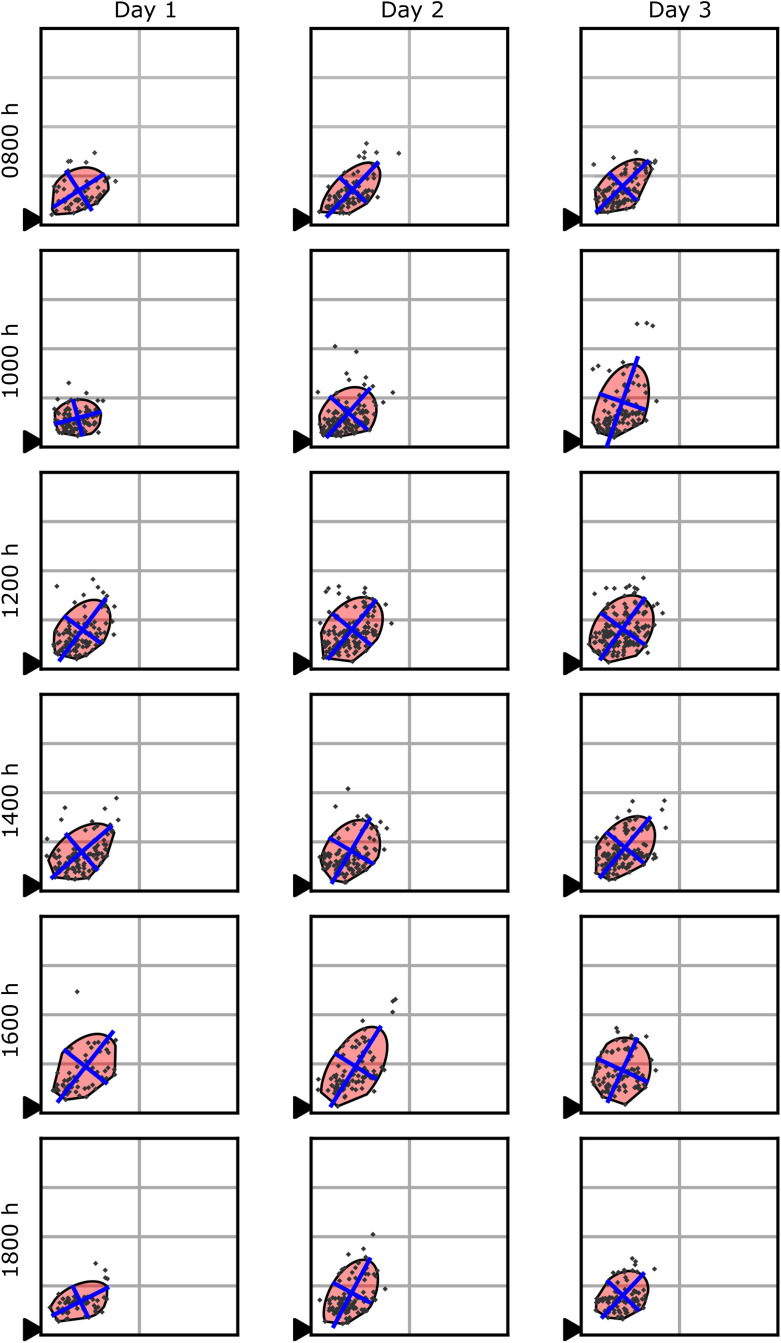
Dance floors in Colony 1 across days and observation times. The 2000-hour observations were omitted to improve readability and because there were only 10, 6, and 6 dances on days 1, 2, and 3, respectively. All eight frames (100 × 100 cm) are shown.

### Dance floor definition

All steps were performed in R version 4.2.1 [21]. Dance floors were defined in three steps: (1) calculating the convex hull, (2) generating a confidence ellipse, and (3) computing their spatial intersection. A minimum of three dances in an observation period were required to calculate a dance floor, as fewer cannot define a two-dimensional polygon. Full implementation details and annotated code for all steps are available in the Figshare repository (https://doi.org/10.6084/m9.figshare.29483207).

### Convex hull

The convex hull is the smallest convex polygon that encloses all dance locations from a given observation, analogous to stretching an elastic band around the outermost points

(Fig 1A). For each observation period, we used the base R function chull() to calculate the convex hull around all recorded dance coordinates to define the outer boundary of the distribution. This provided an inclusive boundary capturing all dances, including peripheral or outlying points.

### Confidence ellipse

To complement the convex hull and limit the influence of outliers, we also calculated a confidence ellipse based on the spatial distribution of dance coordinates, similar in concept to the elliptical region generated by Wario et al. [20]. We first computed the mean location and covariance matrix of the dance coordinates using the base R function cov(). From the covariance matrix, we extracted the major and minor axes of variation and their orientation via eigen decomposition using the base R function eigen(). The ellipse was centred on the mean location, with axes scaled to two standard deviations (2-SD), which captures the core spatial extent of the distribution while limiting the influence of peripheral or isolated points (Fig 1A). Under a bivariate normal distribution, a 2-SD ellipse would contain approximately 95% of points; in practice, this proportion varied due to skew and spatial constraints imposed by hive walls.

### Spatial intersection

We defined the dance floor as the spatial intersection of the convex hull and the 2-SD confidence ellipse (Fig 1B). To enable spatial intersection, both the convex hull and the confidence ellipse (approximated as a polygon) were converted to spatial objects using the sf package version 1.0.15 [22,23], and their overlap was computed geometrically. Intersection of the two objects was performed with the sf function st_intersection(). Convex hull vertices that fell within the confidence ellipse were included. The intersection was used to minimize excess area when a small number of dances occurred far from the main cluster of dances, when the dances were not distributed normally, or when confidence ellipses lie adjacent to or extend beyond the edges of the hive. This approach allowed us to focus on the central cluster of dances while excluding extreme or isolated dances that might distort measures of area, orientation, or centroid location. Dances outside the confidence ellipse were excluded by the intersection, effectively treating them as outliers. This approach ensured the dance floor captured both central tendency and boundary limits of dance distributions while excluding extreme outliers.

### Dance floor metrics

From each dance floor polygon, we extracted seven metrics: *Area* and *Perimeter* (in cm^2^ and cm), *X* and *Y* (horizontal and vertical coordinates of the centroid relative to the hive entrance, in cm), *Length* and *Width* (major and minor axes of the confidence ellipse, in cm), and *Angle* (orientation of the major axis in degrees from horizontal) (Fig 1B). *Area*, *X*, and *Y* were calculated with the sf functions st_area() and st_centroid(). *Perimeter* was calculated by casting the dance floor polygon to a MULTILINESTRING object with st_cast() and summing edge lengths using st_length() (both from the package sf). These metrics provided quantitative descriptors of the dance floor’s size, position, and spatial configuration.

### Visualization

**Fig 4:**
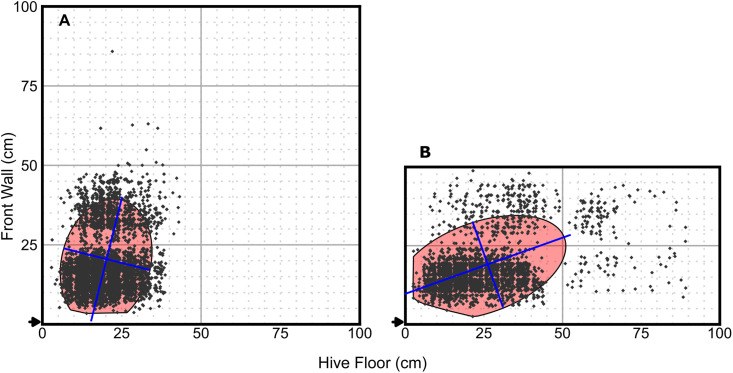
Cumulative dance floor regions for all 8-frame and 4-frame hives. (A) Each polygon represents the ellipse intersection calculated from all waggle dances across all days, times, and colonies for 8-frame hives. (B) Each polygon represents the equivalent cumulative region for 4-frame hives. These footprints illustrate the method’s adaptability to long-term or aggregate spatial patterns but were not used for statistical analysis in the present report. Dance floor metrics are listed in supplemental S1 Table. Note the lack of dances on and near the wooden boundaries of the frames of the beehive (at *x* = 50 cm and at *y* = 25 cm). Note also the perceived lack of dances at each of the 5 × 5-cm grid lines due to observers marking dances beside grid lines on the printed maps rather than on the grid lines.

For visual evaluation (e.g., Figs 1–4), we plotted the dance floor boundary, the major and minor axes of the confidence ellipse (recentred at the dance floor centroid), and the observed dance locations. These plots enabled visual inspection of dance floor shape, size, position, and angle across days and hive sizes. Fig 2 demonstrates a comparison of the dance floors from three different 30-min observation periods of one day from two different colonies. Fig 3 illustrates dance floors from a single colony over several days and observation periods. Fig 4 illustrates a cumulative dance floor constructed by aggregating all waggle dances across observation periods and colonies for each hive size. (Fig 4 demonstrates an alternative use of our method to visualize long-term spatial patterns; the calculated dance floors are aggregates of multiple observation periods and are not those used in the statistical analyses below.)

**Fig 5:**
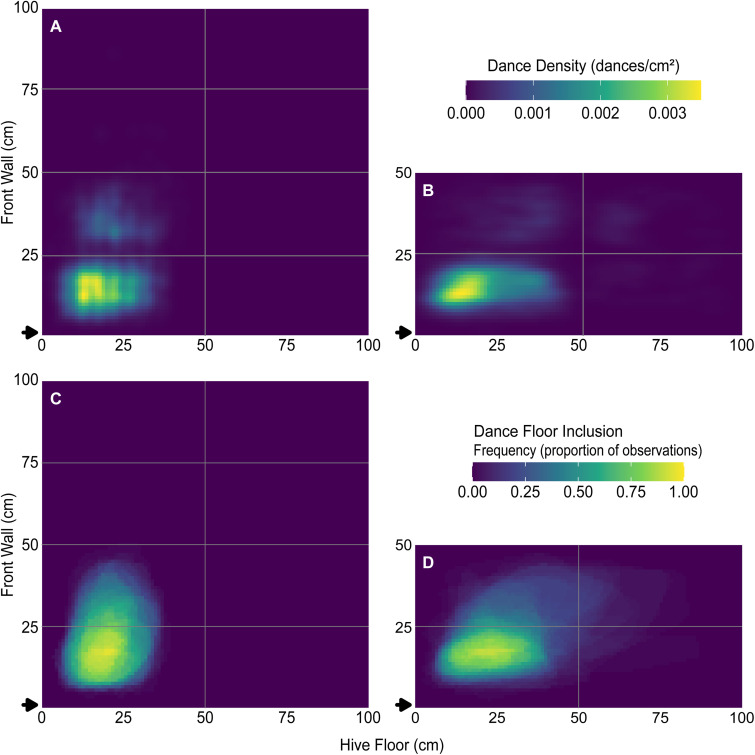
Dance-density and dance floor-inclusion heatmaps. Dance densities were calculated from all observation periods over all days from (A) Colonies 1–4 (8-frame) and (B) Colonies 5–8 (4-frame). Note that the lack of dances on the wooden frame boundaries and the perceived lack of dances on the grid lines are still visible in panel A (see Fig 4). Dance floor-inclusions were calculated for each point in the hive as the proportion of observations that are included in the calculated dance floor from (C) Colonies 1–4 (8-frame) and (D) Colonies 5–8 (4-frame).

To visualize broader spatial patterns, we created two heatmaps: (1) a dance density heatmap showing the smoothed distribution of dances (Fig 5A, B), using two-dimensional kernel density estimation using the function stat_density_2d() from the package ggplot2 version 3.5.1 [24] (a technique that converts discrete points into a continuous density surface [25]), and (2) a dance floor inclusion heatmap (Fig 5C, D), showing the proportion of trials in which each 1 cm^2^ grid cell fell within a dance floor. Both heatmaps were rendered at 1 cm^2^ resolution.

### Statistical analyses

Although our method can be applied to any subset of dances—such as daily or trial-wide aggregates (e.g., Fig 4)—here, we computed and analyzed dance floors at the level of individual 30-minute observation periods (e.g., Figs 1–3). This approach preserved temporal resolution and allowed testing for systematic variation across time and conditions. To confirm that the method performed consistently across hive sizes, we constructed a linear model using the base R function lm() to compare the proportion of dances captured by the defined dance floors between 4-frame and 8-frame hives. We then used the function cor() from the base package stats to identify and remove highly correlated predictors (|*r*| > 0.9) before constructing univariate mixed-effects models for each metric and a multivariate model to assess overall configuration. All analyses were performed in R using a significance level of *α* = 0.05. Full implementation details are available in the Figshare repository.

### Univariate analyses

To investigate how honey bee dance floor characteristics varied across contexts, we constructed linear mixed-effects models for each of the dependent variables described above using the lmer() function from the package lmerTest version 3.1.3, which extends lme4 version 1.1.31 [26,27] To determine whether *Colony* should be included as a random effect, we used likelihood ratio tests (LRTs) to compare models with and without the random term, both fit using maximum likelihood (ML) to permit valid comparisons. Fixed effects were selected from among *Time* (number of hours since sunrise), *TrialDay* (the sequential day number of the trial, 1–5), and *DOY* (day of year, the number of days since January 1), as well as their interactions with *Size* of the hive (4-frame or 8-frame), which we treated as a central explanatory variable.

For each dependent variable, we began with a full model including all main effects and interactions with *Size*. We used the drop1() function from the base package stats (with Satterthwaite’s method) to identify the least informative interaction terms based on F-tests and iteratively removed them to generate a sequence of nested candidate models. At each step, we used both LRT and Akaike information criterion (AIC) comparisons (fit via ML) to assess model fit. We retained a model term if its removal caused either a significant loss of fit (LRT *P* < 0.05) or an increase in AIC of at least 2 units. This combined rule balanced statistical support and parsimony. For each outcome, we retained the simplest model that satisfied these criteria.

### Multivariate analysis

We also constructed a multivariate model incorporating all dependent variables using the function manova() from the base package stats. We compared the full model (including all main effects and *Size* interactions) to progressively simplified models using Pillai’s trace, the default test statistic in MANOVA output. *Colony* was included as a random intercept in all univariate models to account for repeated measures, but it was not included as a fixed effect in the MANOVA models. This decision was based on complete confounding between *Colony* and *Size*: Colonies 1–4 were in 8-frame hives, and Colonies 5–8 in 4-frame hives (Table 1). As a result, including both *Colony* and *Size* as fixed effects would introduce perfect multicollinearity, making it impossible to estimate their independent contributions. We therefore retained *Size*—our primary explanatory variable of interest—and omitted *Colony* to preserve model interpretability.

## Results

We defined the dance floor for each 30-min observation as the intersection of the convex hull and the 2-SD confidence ellipse of observed dance locations. Most dance floors were more-or-less elliptical and generally located near the hive entrance. Descriptive statistics for the seven dance floor metrics—*Area*, *Perimeter*, *Length*, *Width*, *Angle*, and centroid *X* and *Y*—are listed in Table 2. Visualizations of average dance density (Fig 5A, B) and consistency of dance floor locations across colonies (Fig 5C, D) illustrate both the spatial compactness and temporal stability of the defined dance floor regions.

**Table 2:** Mean values of the seven dance floor metrics.

Metric	Mean ± SD
*Area* (cm^2^)	513.2 ± 312.2
*Perimeter* (cm)	88.6 ± 27.3
*Length* (major axis of confidence ellipse) (cm)	43.9 ± 16.8
*Width* (minor axis of confidence ellipse) (cm)	21.1 ± 7.8
*Angle* (of major axis from horizontal) (°)	14.7 ± 57.2
*X* (horizontal distance of centroid to hive entrance) (cm)	24.0 ± 6.1
*Y* (vertical distance of centroid to hive entrance) (cm)	20.5 ± 5.2

Means ± standard deviations for all colonies, hive sizes, days, and observations times are shown (*n* = 155 calculated dance floors). Although 189 observations were completed, only 155 met the inclusion criterion of at least three waggle dances per observation.

All seven metrics were approximately normally distributed, and residual diagnostics confirmed that model assumptions were reasonably met without the need for transformation. The metrics *Area* and *Perimeter* were tightly correlated (Pearson *r*_153_ = 0.92, *P* < 0.0001), likely due to the defined dance floors being convex in shape and thus lacking long extensions or irregular protrusions. We therefore retained *Area* and excluded *Perimeter* from all subsequent analyses. All other pairwise correlations were more moderate (|*r*| < 0.85).

**Fig 6:**
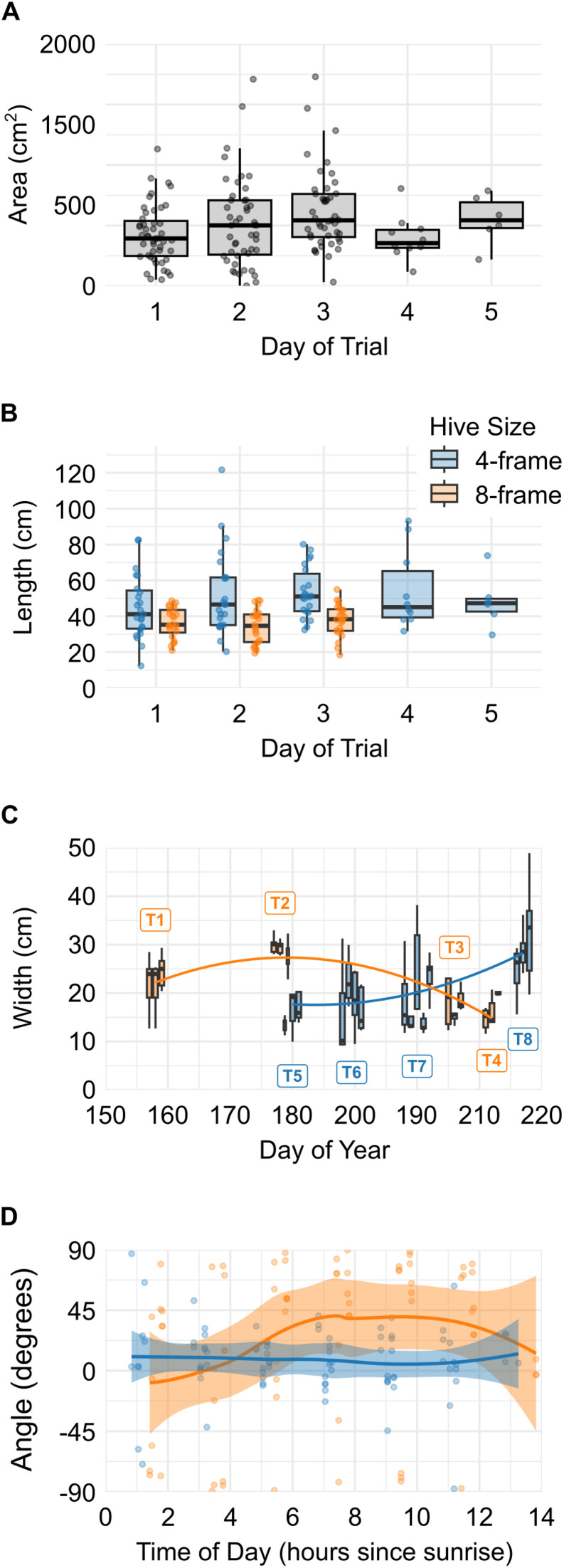
Summary of the most informative relationships among the four shape parameters. (A) *Area* varied significantly by *TrialDay*, though no consistent pattern was evident across days. (B) *Length* varied significantly with both *TrialDay* and hive *Size*; 4-frame hives generally showed longer dance floors than 8-frame hives. (C) *Width* exhibited a significant interaction between hive *Size* and *DOY*: in 8-frame hives, width peaked mid-season before declining, whereas in 4-frame hives, width was lowest mid-season and increased late in the season. Boxplots represent daily distributions; overlaid curves are quadratic regressions of colony-level means at the median day of each colony. Labels T1–T8 identify the colonies (trials). (D) Dance floor *Angle* was influenced by a significant *Size* × *Time* interaction: in 4-frame hives, orientation remained relatively stable throughout the day, while in 8-frame hives, angle varied more strongly with time. Loess smoothing curves with 95% confidence intervals are shown. Colour key (panel B) applies to panels B–D.

**Fig 7:**
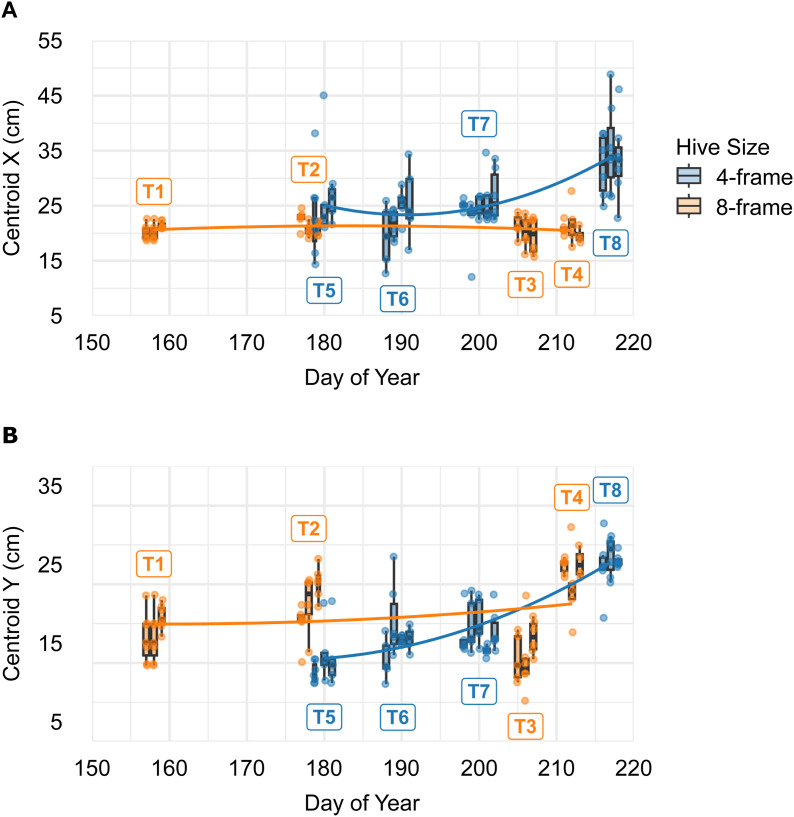
Summary of spatial dynamics in dance floor location. (A) *X* (horizontal location) of the dance floor centroid as a function of *DOY* and hive *Size*. Both main effects and their interaction were significant. In 8-frame hives, *X* remained relatively stable throughout the season, whereas in 4-frame hives, horizontal position varied over time. (B) *Y* (vertical location) of the centroid modelled using a full fixed-effects model that included *Size* and its interactions with *Time*, *DOY*, and *TrialDay*. All terms were significant except for *Time* and its interaction with *Size*. *Y* remained stable in 8-frame hives but increased across the season in 4-frame hives. In both panels, quadratic regressions were fit to colony-level means plotted at the median day of each trial. Labels T1–T8 identify the colonies (trials).

We analyzed each metric using univariate linear mixed-effects models and assessed the overall structure of the dance floor with a MANOVA. Together, these approaches provided complementary insights into how dance floor characteristics varied across colonies, particularly in relation to hive *Size* and day of year (*DOY*) (Table 3). We first describe how the predictor variables influenced the shape of the dance floor (*Area*, *Length*, *Width*, and *Angle*), followed by their effects on dance floor position (centroid *X* and *Y*). For clarity and interpretability, we focused the main visualizations on the most informative effects, as these offered the clearest insights into how dance floor structure varied across contexts. All seven metrics (including *Perimeter*) were visualized against the four key predictors in a series of supplemental figures (S1–S7 Figs). The most informative relationships are shown in Figs 6 and 7, with full statistical results listed in Table 3.

**Table 3:** Results of the univariate (*Area*, *Length*, *Width*, *Angle*, *X*, and *Y*) and multivariate models.

Model ^*a*^	Formula ^*b*^	Significant Fixed Effects	LRT ^*c*^
*Area*	*TrialDay* + (1 | *Colony*)	*TrialDay*: *F*_1, 149.43_ = 13.38, *P* = 0.0004	χ^2^_1_ = 23.1, *P* < 0.0001
*Length*	*TrialDay* + *Size* + (1 | *Colony*)	*TrialDay*: *F*_1, 151.28_ = 7.71, *P* = 0.0062 *Size*: *F*_1, 8.12_ = 5.87, *P* = 0.0413	χ^2^_1_ = 11.9, *P* = 0.0006
*Width*	*DOY* × *Size* + (1 | *Colony*)	*Size*: *F*_1, 8.29_ = 16.56, *P* = 0.0033 *Size* × *DOY*: *F*_1, 8.30_ = 16.75, *P* = 0.0032	χ^2^_1_ = 7.3, *P* = 0.0069
*Angle*	*Time* × *Size +* (1 | *Colony*)	*Size* × *Time*: *F*_1, 148.34_ = 5.36, *P* = 0.0220	χ^2^_1_ = 6.1, *P* = 0.0132
*X*	*DOY* × *Size* + (1 | *Colony*)	*DOY*: *F*_1, 8.57_ = 12.90, *P* = 0.0063 *Size*: *F*_1, 8.55_ = 11.28, *P* = 0.0090 *Size* × *DOY*: *F*_1, 8.57_ = 13.93, *P* = 0.0051	χ^2^_1_ = 8.8, *P* = 0.0031
*Y*	(*Time* + *TrialDay* + *DOY*) × *Size* + (1 | *Colony*)	*TrialDay*: *F*_1, 154.99_ = 4.20, *P* = 0.0422 *DOY*: *F*_1, 7.89_ = 10.84, *P* = 0.0112 *Size*: *F*_1, 7.96_ = 5.38, *P* = 0.0492 *Size* × *TrialDay*: *F*_1, 154.99_ = 7.76, *P* = 0.0060 *Size* × *DOY*: *F*_1, 7.89_ = 5.80, *P* = 0.0431	χ^2^_1_ = 62.1, *P* < 0.0001
MANOVA	(*Time* + *TrialDay* + *DOY*) × *Size*	*DOY*: *Pillai* = 0.42, *F*_6, 142_ = 17.18, *P* < 0.0001 *Size*: *Pillai* = 0.37, *F*_6, 142_ = 14.00, *P* < 0.0001 *Size* × *DOY*: *Pillai* = 0.44, *F*_6, 142_ = 18.53, *P* < 0.0001 *Size* × *Time*: *Pillai* = 0.10, *F*_6, 142_ = 2.59, *P* = 0.0208	n/a

^a^Univariate mixed-effects models for the dependent variables were evaluated with the R function lmer() from package lmerTest. The multivariate model on all six dependent variables was evaluated with the R function manova().

^b^The simplified model formulae after non-contributing terms were eliminated. In all univariate models, *Colony* was treated as random effect.

^c^Likelihood ratio test (LRT) comparing models with and without *Colony* as a random intercept. In all cases, including *Colony* improved the model.

The shape of the dance floor was influenced by multiple predictors, with distinct patterns emerging for *Length*, *Width*, and *Angle*. *Area* differed significantly across days of the trial, but the variation did not follow a consistent trend (Fig 6A). *Length* was significantly affected by both *TrialDay* and *Size*, with dance floors tending to be longer on later days and, interestingly, in smaller hives (Fig 6B). *Width* exhibited a significant *Size* × *DOY* interaction, indicating that seasonal changes in dance floor width differed between hive sizes. In 8-frame hives, *Width* was relatively high early in the season and dropped later in the season. In 4-frame hives, *Width* remained relatively low through the middle of the season and increased late in the season (Fig 6C). The only shape parameter influenced by *Time* was *Angle*, which showed a hive *Size*-specific temporal trend: while orientation remained stable throughout the day in 4-frame hives, it shifted over time in 8-frame hives (Fig 6D). An anecdotal example of *Angle* changing differently in different size hives can also be seen in Fig 2.

The position of the dance floor centroid also varied across colonies, particularly in relation to *Size* and *DOY*. Both centroid *X* and *Y* were significantly affected by *DOY*, *Size*, and their interaction. In 4-frame hives, the *X*-coordinate of the centroid tended to shift rightward later in the season, whereas in 8-frame hives, it remained relatively stable (Fig 7A). Similarly, the vertical position (*Y*) rose over time in 4-frame hives but was more consistent in 8-frame hives (Fig 7B). These *Size*-specific seasonal trajectories suggest that the smaller colonies adjusted the placement of their dance activity more dynamically over time, shifting it further away from the hive entrance later in the season. In spatial terms, these seasonal shifts represented only a small fraction of the available comb area in 8-frame hives but a substantially larger relocation of the dance floor in 4-frame hives.

**Fig 8:**
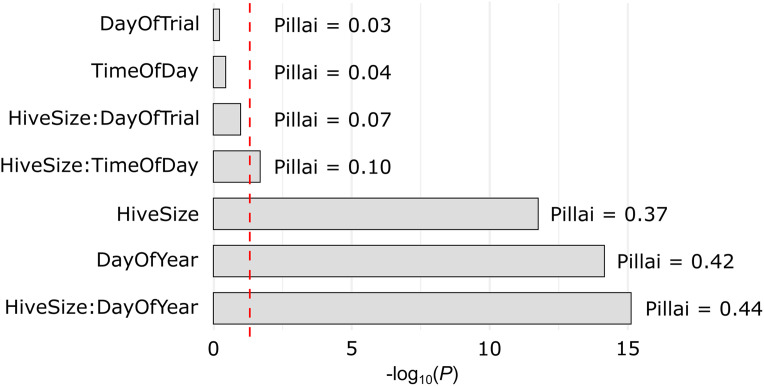
Multivariate effects of key predictors on overall dance floor structure. Bars show –log₁₀(*P*) from the MANOVA, with higher values indicating greater significance. Text labels denote the corresponding Pillai’s trace values. The red dashed line indicates the *P* = 0.05 threshold (–log₁₀(0.05) ≈ 1.3). Hive *Size*, *DOY*, and their interaction explained the greatest multivariate variation, followed by a weaker but significant effect of the interaction of hive *Size* and *Time* of day. All other factors had minimal effect.

To further assess how dance floor structure varied across colonies and contexts, we used a multivariate linear model incorporating *Time*, *TrialDay*, and *DOY*, along with their interactions with hive *Size*. Pillai’s trace indicated that the full model provided a significantly better fit than a main-effects-only model (Pillai’s trace = 0.587, *F*_18, 432_ = 5.84, *P* < 0.0001) and was thus selected for the MANOVA test. Based on multivariate F-tests (Table 3), *Size*, *DOY*, and their interaction all had strong and significant effects on dance floor structure, while *Size* × *Time* was also significant but weaker. These results are visualized in Fig 8, which displays all terms in the model for comparison, including those that did not reach significance. The MANOVA results align in part with the univariate findings, identifying *DOY*, *Size*, and their interaction as significant contributors to variation in dance floor structure. These multivariate effects echo key univariate trends, particularly in *Width*, *X*, and *Y*, but also capture broader covariance patterns not fully reflected in any single response variable. This pattern suggests that some predictors (e.g., *Size* × *DOY*) influence multiple traits in concert, while others have more localized, trait-specific effects.

Finally, our dance floor definition consistently captured the majority of waggle dances across all colonies, with a mean inclusion rate of 90.8% ± 4.9% SD (S8 Fig), further supporting its robustness and generalizability across contexts. A linear model revealed no significant difference in capture rate between hive sizes (*F*_1, 153_ = 0.185, *P* = 0.668), confirming that the dance floor definition was equally applicable across experimental contexts.

## Discussion

Our geometric definition of the honey bee dance floor offers a new framework for quantifying spatial organization of recruitment dances. It yields distinct metrics that can be compared across time of day or time of year, among colonies, or across ecological contexts. Applying this method, we revealed that the spatial structure of the dance floor varied in systematic ways across time and hive conditions.

For example, in these demonstration datasets, orientation tended to shift across the day, and centroid position varied with season and hive configuration, illustrating the framework’s ability to detect biologically meaningful spatial patterns. Dance floor area, length, and vertical position of the centroid each changed significantly over the course of the trials, suggesting gradual temporal shifts in spatial use. Changes in area were driven only by trial day rather than season or hive size, yet it did not follow a consistent pattern throughout the season (Fig 6A). This likely reflects the fact that each trial captured only a short window of ongoing colony activity, making dance floor area a more volatile or context-dependent measure. Dance floor length (major axis) also changed over trial days and was greater in 4-frame hives, with this size effect consistent across the season (Fig 6B). The vertical position of the dance floor centroid was particularly dynamic, varying with trial day, day of year, and hive size, as well as in interactions involving hive size and both time variables (Fig 7B). These relationships indicated that small and large hives adjusted vertical dance locations differently over different time scales, consistent with prior observations showing that dance behaviour is flexible and responsive to changes in colony structure, crowding, and environmental context [3,15,16].

Dance floor width and both the horizontal and vertical positions of the dance floor centroid were affected by season (day of year), although the effect on width was only through interaction with the size of the hive: widths in 8-frame hives peaked early and declined over time, while widths in 4-frame hives increased steadily as the season progressed (Fig 6C). This unexpected divergence suggests that smaller and larger colonies reorganize space differently in response to seasonal pressures. Like the vertical position of the dance floor centroid, the horizontal position was also affected by season, as well as by hive size and their interaction (Fig 7A). However, these were the only variables that affected the horizontal position.

Dance floor orientation (*Angle*) was the only spatial metric to show a significant effect of time of day, and it did so in interaction with hive size. In 8-frame hives, angle shifted markedly throughout the day, increasing during the morning and declining in the afternoon, while in 4-frame hives, it remained consistently aligned near zero (Fig 6D). This pattern may reflect spatial constraints in smaller hives, where limited comb area restricts the ability of foragers to reorganize the dance floor as colony activity builds. In contrast, larger hives appear to support more flexible within-day reorientations, possibly in response to changing traffic patterns or crowding dynamics. While the shift in orientation might suggest a relationship to the sun’s changing position across the sky, the symmetric rise and fall across midday does not match the continuous change in solar azimuth and is therefore unlikely to reflect celestial alignment alone.

Although the horizontal and vertical centroid positions followed similar visual trajectories, their correlation was modest (*r* = 0.32), indicating that shifts in these axes were only partially coupled. This may reflect the fact that the vertical position in 4-frame hives remained stable for part of the season before rising, while horizontal position increased more steadily. Time of day did not affect centroid position in either axis, however, aligning in part with prior findings from Ortis et al. [19], who reported no time-of-day effects on dance location. Our results extend this, showing that positional shifts were modulated by seasonal and colony-level factors rather than the daily cycle. Also notable is that interaction effects varied across traits: centroid position (*X* and *Y*) shifted primarily in 4-frame hives, whereas angle shifted across time of day only in 8-frame hives. This asymmetry suggests that the processes governing long-term spatial displacement and short-term reorientation may rely on distinct constraints, such as crowding, comb layout, or internal traffic patterns, and cannot be attributed to available space alone.

The MANOVA results partially mirrored the univariate findings, identifying day of year, hive size, and their interaction as significant predictors of overall dance floor structure when considered as a multivariate trait. Notably, the MANOVA also identified a significant interaction between hive size and time of day, despite this effect being absent from most univariate models. This suggests that subtle, coordinated shifts in spatial organization may occur throughout the day, even if they are not strongly expressed in any single metric. While not all univariate effects were reflected in the multivariate model, the MANOVA confirms that these predictors influenced the spatial organization of recruitment activity in a coordinated manner across multiple traits. This reinforces the interpretation that seasonal and contextual factors shape the dance floor as an integrated system, not merely as a collection of independent spatial metrics.

The heatmaps in Fig 5 further illustrate the spatial compactness and consistency of dance activity across colonies. Despite differences in hive size and setting, both dance density and inclusion frequency were concentrated within a relatively constrained region near the centre of the frame closest to the hive entrance, confirming the tendency of foragers to restrict recruitment activity to a stable location within the hive. The absence of dancing at frame edges and the visible effect of the wooden boundaries of the frames underscore how physical features of the comb or observation environment can influence recorded dance locations. Importantly, the regions captured by our calculated dance floor definitions align well with these high-density zones, e.g., the findings of Seeley and Towne [15], further validating our geometric approach to defining the dance floor.

Hive size emerged as the most consistently important predictor in our analyses, significantly influencing four individual metrics—length, width, and both horizontal and vertical centroid positions—as well as overall spatial structure in the MANOVA. It also interacted with other key variables, including day of year, trial day, and time of day, suggesting that colony size shaped not just the average spatial organization of waggle dancing, but also how that organization changed over time. Although hive size did not affect every spatial parameter—such as area or angle—it consistently contributed to variation across multiple dimensions of dance floor structure, highlighting its consistent source of variation in regulating recruitment behaviour. It should be noted, however, that all 8-frame-hive experiments were performed in 2012 in a shed in an open field, and all 4-frame-hive experiments were performed in 2013 in a laboratory on a university campus. Thus, we cannot say with certainty whether differences between 8-frame and 4-frame hives reflect hive size itself, or the confounded effects of hive size, year, location, and ambient conditions. Because this study’s aim was to demonstrate and validate a quantitative method rather than to test specific biological hypotheses, these confounding factors do not affect our primary conclusions. Future studies using this framework could explicitly vary hive size or environmental conditions to assess their independent contributions. Overall, our spatial definition consistently captured the majority of waggle dances across all colonies, reinforcing its robustness and generalizability across ecological contexts.

Trial day also influenced several aspects of dance floor structure, including area, length, and vertical position of the centroid. These effects likely reflect short-term internal dynamics within the colony—such as shifting brood placement, comb restructuring, or changes in forager activity—that unfold even over a few consecutive days. Unlike day of year or hive size, however, trial day was not involved in any strong seasonal or multivariate patterns, and its effects were not always directional (e.g., Fig 6A). This suggests that while short-term internal processes can influence spatial use, they do so in variable, context-dependent ways.

Although the mechanisms guiding where returning foragers choose to dance remain unclear, our findings demonstrate that dance floor structure is shaped in predictable ways by colony and seasonal context. Dances generally occur near the hive entrance [1,8,9,11–13,15] and are probably associated with chemical cues laid down by other foragers [16–18]. Foragers appear to integrate this information with a preference for dancing on brood cells [19]. The nature of the dances themselves likely affects the shape and location of the dance floor. Bees do not remain in a single location while dancing but rather traverse across the comb, and this drift causes dances for more distant foraging sources to be performed further from the hive entrance than dances for nearer sources [1,14,19]. In fact, because the drift may occur in different directions in part due to the direction of the waggle runs of the dances, the dance floor is partially compartmentalized, with different but overlapping regions representing different foraging sources [28]. Additionally, this dance communication system is inherently noisy, exhibiting measurable intra-dance variability in dance angle of up to 15° and in dance duration by as much as 15% within a single dance bout [29–32].

In addition to where foragers choose to dance, whether the foragers choose to dance may also affect the size or shape of the dance floor. Foragers do not always perform recruitment dances for their sources; they are inclined to do so only for valuable sources [14,33]. As foraging sources are ephemeral, their availability and even their relative values change over time. These changes may change the likelihood of foragers choosing to advertise for them. Because dance locations for different sources may form different compartments of the dance floor [28], as bees choose to dance for them or not, the compartments coming and going offline may change the shape of the dance floor. Also, the likelihood of bees dancing for their sources is at least in part a function of how well provisioned the colony is [34]. Thus, the amount of stored honey in the nest may affect the number of bees performing waggle dances, which may alter dance floor size by virtue of how crowded the dance floor is.

It is also interesting to consider potential recruits’ behaviour on the dance floor. For instance, although foragers appear to prefer to dance on brood cells, whether they are open or capped [19], dance followers are more attracted to dances performed on open, empty cells, and such dances are thus more successful in recruiting followers to the advertised sources [6,35]. More broadly, follower attention and recruitment success are influenced by multiple factors, including signal precision, forager experience, and colony context [36,37]. Although these processes shape how information spreads within the colony, our focus here is limited to the spatial organization of dance activity itself.

While our study revealed clear differences in dance floor structure across hive conditions, several limitations should be noted. As stated above, hive size in our study was confounded with both year and experimental location, so we cannot definitively attribute all observed differences to hive size alone, though our methods remain valid for detecting structural variation regardless of its source. Observation hives are also typically smaller than commercial (Langstroth) hives. Even our larger observation hives (8-frame) offered approximately 20,000 cm^2^ of comb area (counting both sides), whereas standard commercial setups typically include two deep boxes, each at least that size, plus additional honey supers [38]. Additionally, our use of unmarked bees prevented us from assessing individual variability or persistence in dance location, which could influence both the structure and stability of the dance floor. Although we modified the observation hives to ensure that all returning foragers danced on the observed face and confirmed through systematic scanning that no dances occurred on the opposite side, entire, unmodified hives could be used instead, resulting in two defined dance floors, one per side. In such cases, dances on the two comb faces would form spatially separate regions that cannot be meaningfully merged into a single planar area; however, the same geometric framework could be applied to quantify each side independently and compare their characteristics if desired. Finally, while our dance floor definition effectively captured the region of waggle dancing activity, it remains a statistical boundary; behavioural validation—e.g., whether recruits behave differently inside versus outside the defined space—would help confirm its functional relevance from the bees’ perspective.

Our approach provides a flexible framework for quantifying dance floor organization, opening a range of possibilities for future research. Although our analyses focused on individual 30-minute timepoints, the same spatial definition can be applied at broader temporal scales. For instance, dance floors could be constructed from all dances performed on a given day or across multiple days to explore longer-term spatial trends. Fig 4 provides one example of such an aggregate footprint, illustrating how the method can be adapted for different experimental questions. Our method could be extended to examine how dance floor structure is influenced by hive architecture, including entrance location, internal layout, and colony population density. Combining spatial dance data with annotated substrate maps—including brood, nectar, and empty cells—would also allow for fine-scale analysis of how bees integrate structural and chemical cues. Environmental context remains another key axis of variation: dance floors could be compared across landscapes, climates, or agricultural systems, or used to track changes in resource availability and colony health. For example, Pusceddu et al. [39] reported more dispersed dances in mite-infested hives, suggesting that dance floor structure might serve as an indirect metric of colony stress. Cross-species comparisons also present a promising direction, as different *Apis* species exhibit systematic differences in dance orientation [40], which may yield distinct spatial footprints. Beyond the waggle dance, our method could be adapted to quantify other spatial nest features, such as the brood nest. Finally, automated detection systems like BeesBook and the Waggle Dance Detector module [41,42] could be integrated to streamline future analyses and reduce observer bias.

As outlined in the Introduction, our proposed definition allowed us to address five central questions about the honey bee dance floor: (i) Does the dance floor change throughout the day? Yes—the angle of the dance floor (the angle of its major axis from horizontal) varied with time of day in interaction with hive size: dance floors in larger hives rotated throughout the day, whereas those in smaller hives remained relatively stable in orientation. (ii) Does the dance floor change from day to day? Yes—trial day was a significant main effect for several dance floor metrics: area, length, and vertical position. This suggests that short-term, cumulative changes within the colony influence dance floor structure. (iii) Do dance floors differ among colonies? Yes—trial identity significantly influenced all individual metrics, and the MANOVA confirmed consistent multivariate differences among colonies. (Each trial was conducted on a different colony.) However, a stronger test of colony-specific effects would involve concurrent trials under matched environmental conditions. (iv) Does season influence the dance floor? Yes—day of year predicted the horizontal and vertical location of the dance floor (*X* and *Y* coordinates of the centroid), and seasonal effects differed by hive size, especially in centroid location and dance floor width. And (v) Does the size of the hive itself influence the dance floor? Yes— hive size was the most consistent main effect across metrics, significantly influencing length, width, and both centroid coordinates. It also interacted with every other predictor variable: time of day, trial day, and day of year. These results demonstrate that the dance floor is shaped by a combination of stable colony-level features and dynamic contextual factors, and they validate the utility of our geometric definition in capturing this variation.

Together, our findings highlight how the structure of the honey bee dance floor reflects both stable colony-level features, such as hive size, and dynamic contextual factors, including time of day and time of year. While individual metrics varied independently in some cases, the MANOVA confirmed that hive size and day of year exerted consistent multivariate effects on overall dance floor structure. Because the framework is independent of hive configuration and data source, it is particularly well suited for future experiments that explicitly manipulate hive size, comb structure, or resource distributions, as well as for planned comparative analyses across species. Importantly, our dance floor definition provided a robust and generalizable framework for quantifying this spatial behaviour. On average, it captured 91% of waggle dances across colonies, closely matching the 94% inclusion rate reported by Seeley and Towne [15] for the area within 24 cm of the hive entrance in a 2-frame observation hive. It also aligns with von Frisch’s early estimate of the dance floor as a region roughly 100 cm^2^ [1]. Although he did not specify whether this estimate referred to one or both sides of the comb, it would indicate the dance floor constituted 2% or 4% of the total 5,000-cm^2^ 2-frame hive. The proportional comb usage in our study (517 cm^2^ of a 10,000- or 20,000-cm^2^ hive, or 2.6–5.2%) falls within a similar range, suggesting functional consistency across hive designs. Whereas Seeley and Towne’s definition was tied to the location of the hive entrance [15], ours was derived directly from behavioural data and adapted flexibly across hive sizes and colony conditions. This data-driven approach offers a scalable tool for future studies of waggle dance localization, enabling consistent comparisons across colonies, environments, and experimental designs.

## Supporting information

S1 Fig Variation in dance floor area across time of day, experimental day, day of year, and hive size. The variable *TrialDay* significantly predicted dance floor area (*P* = 0.0004). Each scatterplot includes a red loess smoothing line with a 95% confidence ribbon (grey). Each boxplot includes individual data points and red dots indicating group means.
(PNG)

S2 Fig Variation in dance floor perimeter across time of day, experimental day, day of year, and hive size. Plot elements are as described in S1 Fig. Note the similarity in all four panels to those of dance floor area (S1 Fig) due to a high correlation between area and perimeter (*r* > 0.92).
(PNG)

S3 Fig Variation in centroid X position of the dance floor across time of day, experimental day, day of year, and hive size. The variables day of year (*P* = 0.0063) and hive size (*P* = 0.0090) significantly predicted horizontal position. Plot elements are as described in S1 Fig.
(PNG)

S4 Fig Variation in centroid Y position of the dance floor across time of day, experimental day, day of year, and hive size. The variables *TrialDay* (*P* = 0.0422), *DOY* (*P* = 0.0112), and *HiveSize* (*P* = 0.0492) significantly predicted vertical position of the dance floor. Plot elements are as described in S1 Fig.
(PNG)

S5 Fig Variation in length of the major axis of the dance floor across time of day, experimental day, day of year, and hive size. The variables *TrialDay* (*P* = 0.0062) and *HiveSize* (*P* = 0.0413) significantly predicted dance floor length. Plot elements are as described in S1 Fig.
(PNG)

S6 Fig Variation in width of the minor axis of the dance floor across time of day, experimental day, day of year, and hive size. Hive size significantly predicted dance floor width (*P* = 0.0033). Plot elements are as described in S1 Fig.
(PNG)

S7 Fig Variation in orientation (angle) of the dance floor across time of day, experimental day, day of year, and hive size. No variables had a main effect on the angle of the major axis of the dance floor. Plot elements are as described in S1 Fig.
(PNG)

S8 Fig Percentage of waggle dances captured by the defined dance floor region. Across all colonies, the dance floor definition consistently captured approximately 91% of waggle dances. There was no significant difference in capture rate between 4-frame and 8-frame hives.
(PNG)

S1 Table Metrics of dance floors illustrated in Figs 1–4. (DOCX)
