# Supplementary figures and images for: Quantifying the honey bee dance floor: A data-driven method for defining and comparing waggle dance regions
Source: PLoS One. 2026 Feb 18;21(2):e0341456. doi: 10.1371/journal.pone.0341456 (PMC12915931; doi:10.1371/journal.pone.0341456)

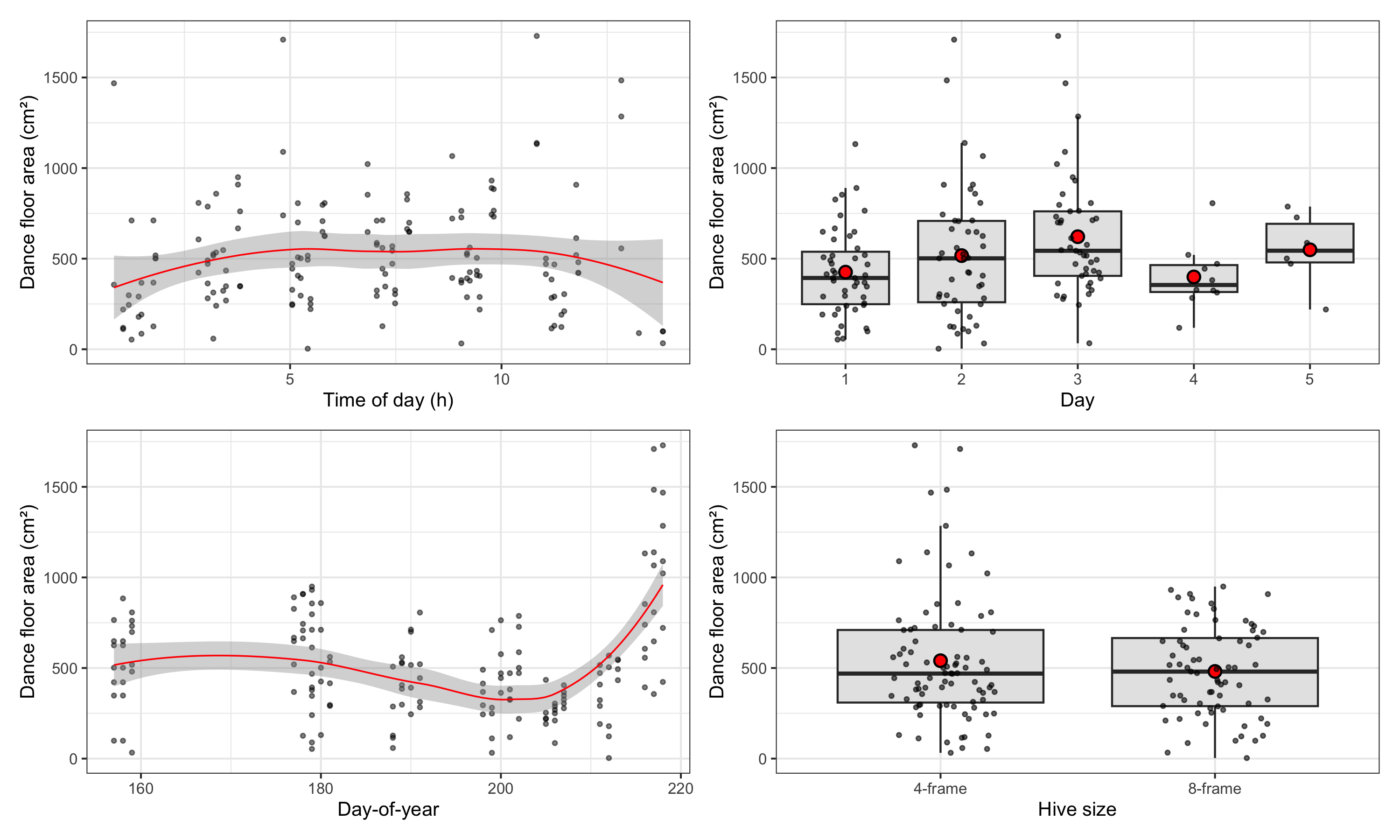

Supplement: S1 Fig — The variable TrialDay significantly predicted dance floor area (P = 0.0004). Each scatterplot includes a red loess smoothing line with a 95% confidence ribbon (grey). Each boxplot includes individual data points and red dots indicating group means. (PNG) [file pone.0341456.s001.png]

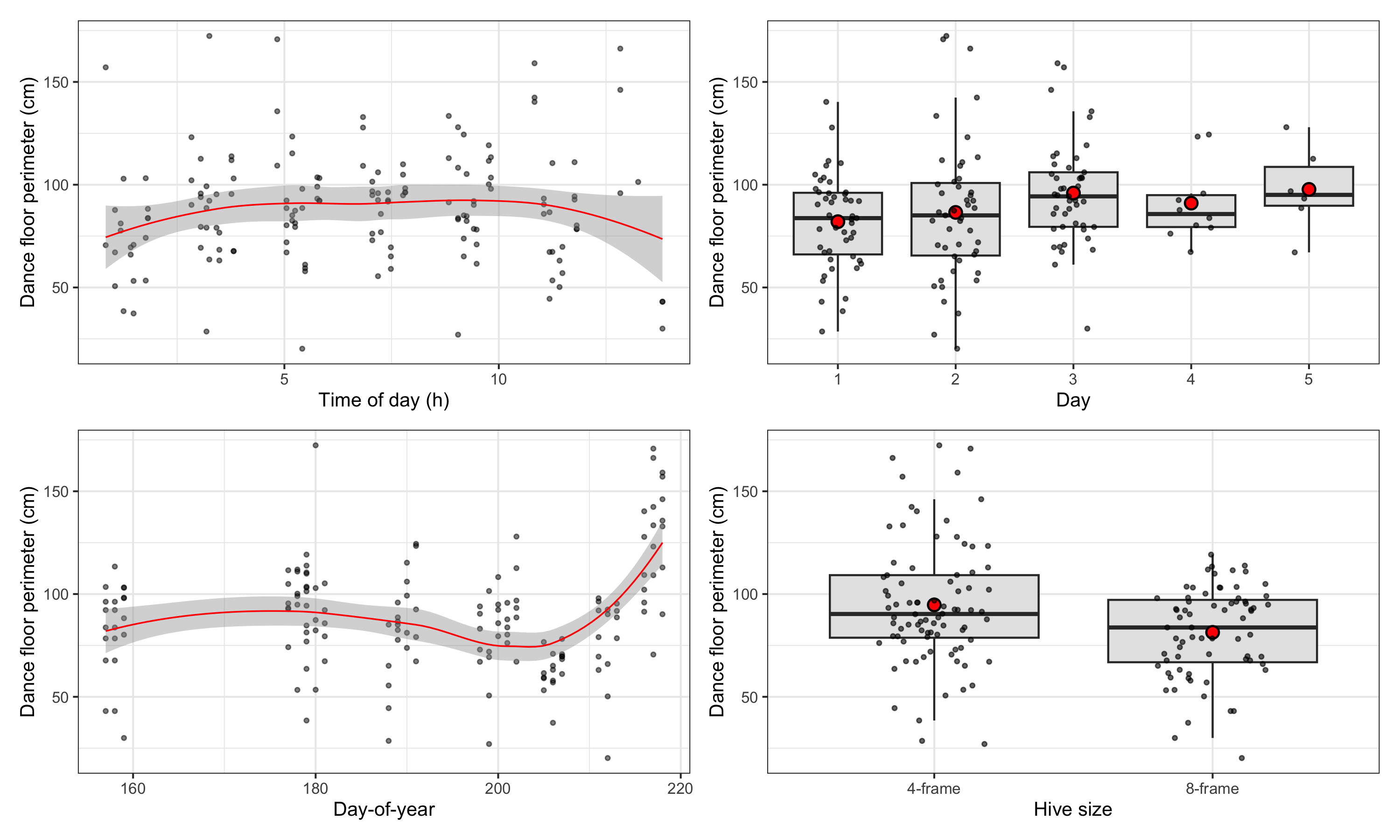

Supplement: S2 Fig — Plot elements are as described in S1 Fig. Note the similarity in all four panels to those of dance floor area (S1 Fig) due to a high correlation between area and perimeter (r > 0.92). (PNG) [file pone.0341456.s002.png]

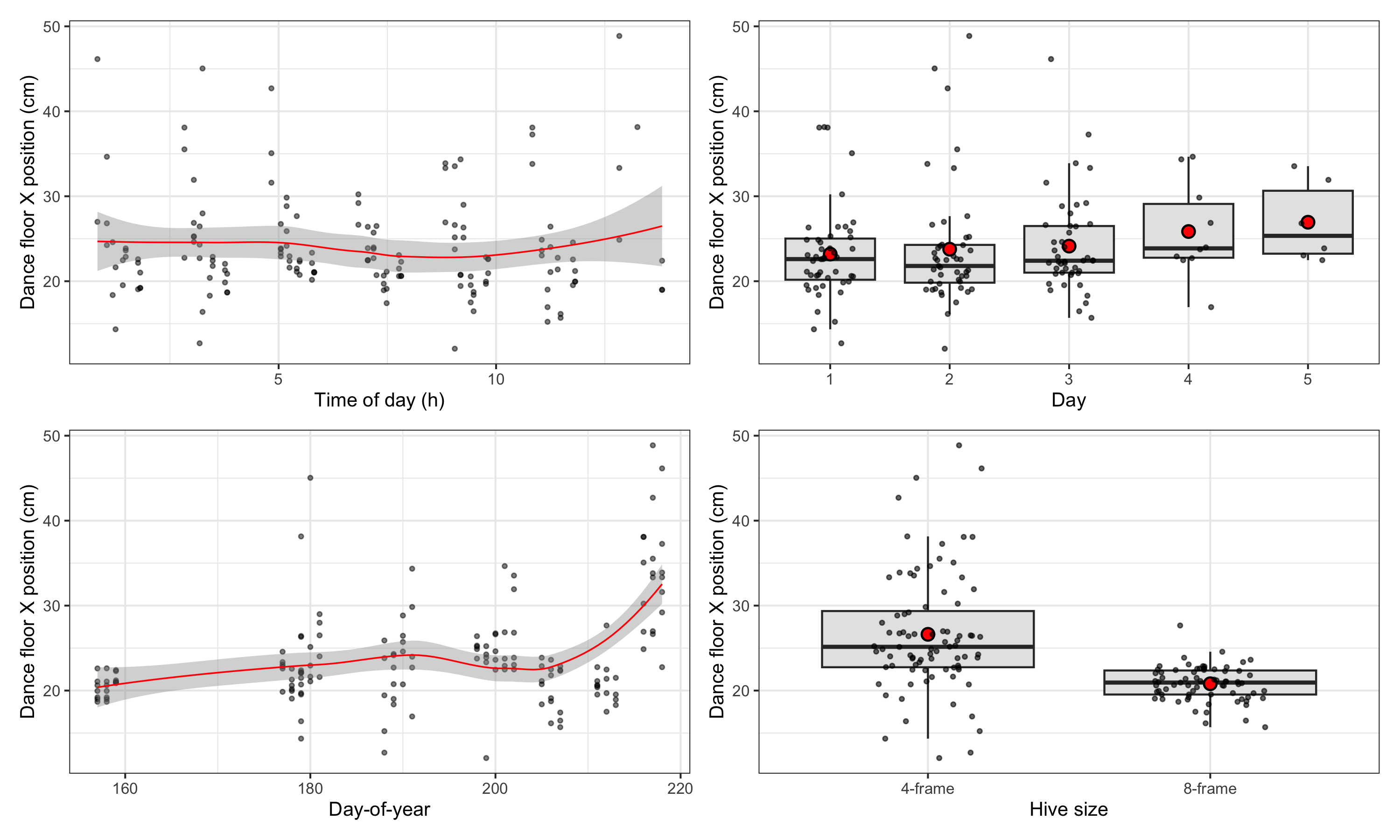

Supplement: S3 Fig — The variables day of year (P = 0.0063) and hive size (P = 0.0090) significantly predicted horizontal position. Plot elements are as described in S1 Fig. (PNG) [file pone.0341456.s003.png]

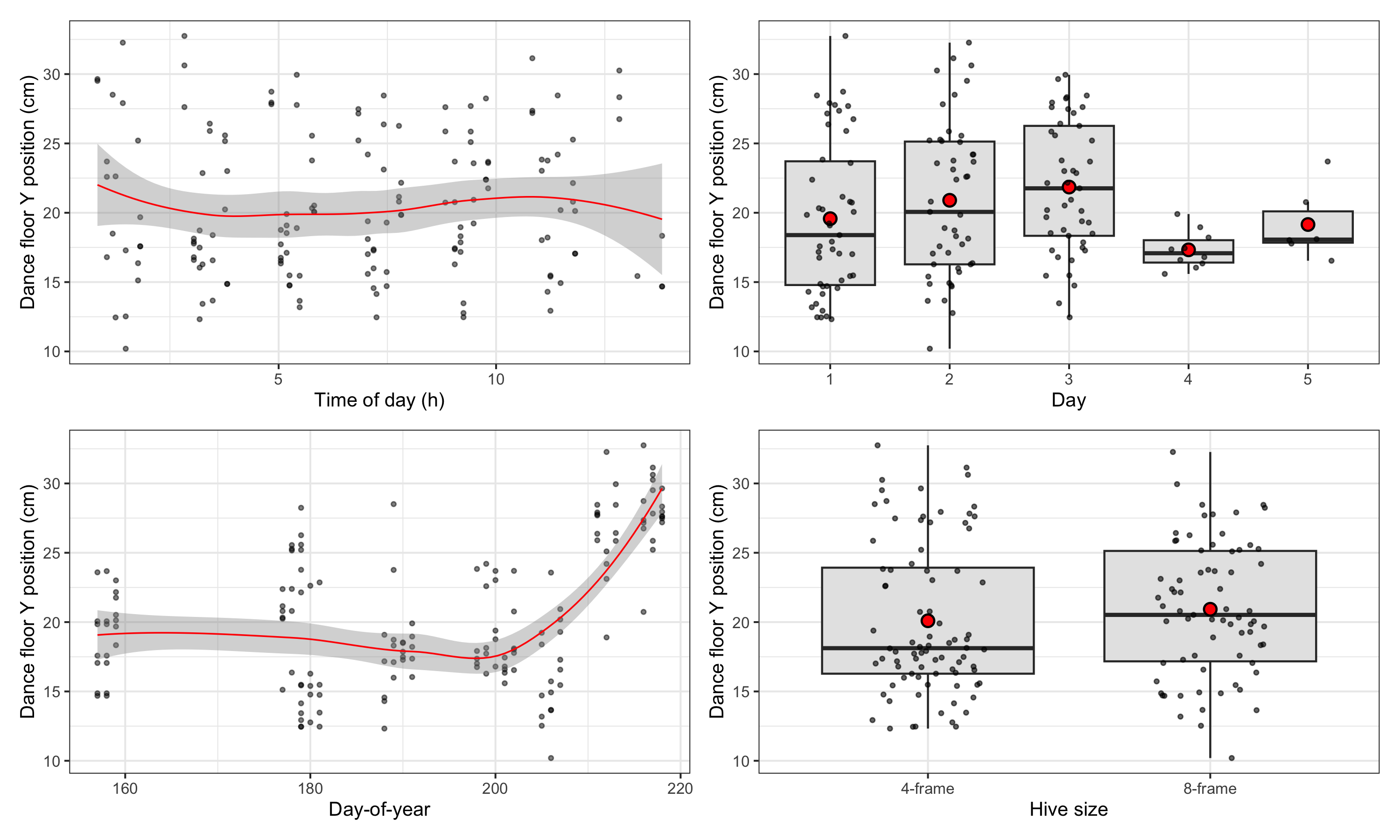

Supplement: S4 Fig — The variables TrialDay (P = 0.0422), DOY (P = 0.0112), and HiveSize (P = 0.0492) significantly predicted vertical position of the dance floor. Plot elements are as described in S1 Fig. (PNG) [file pone.0341456.s004.png]

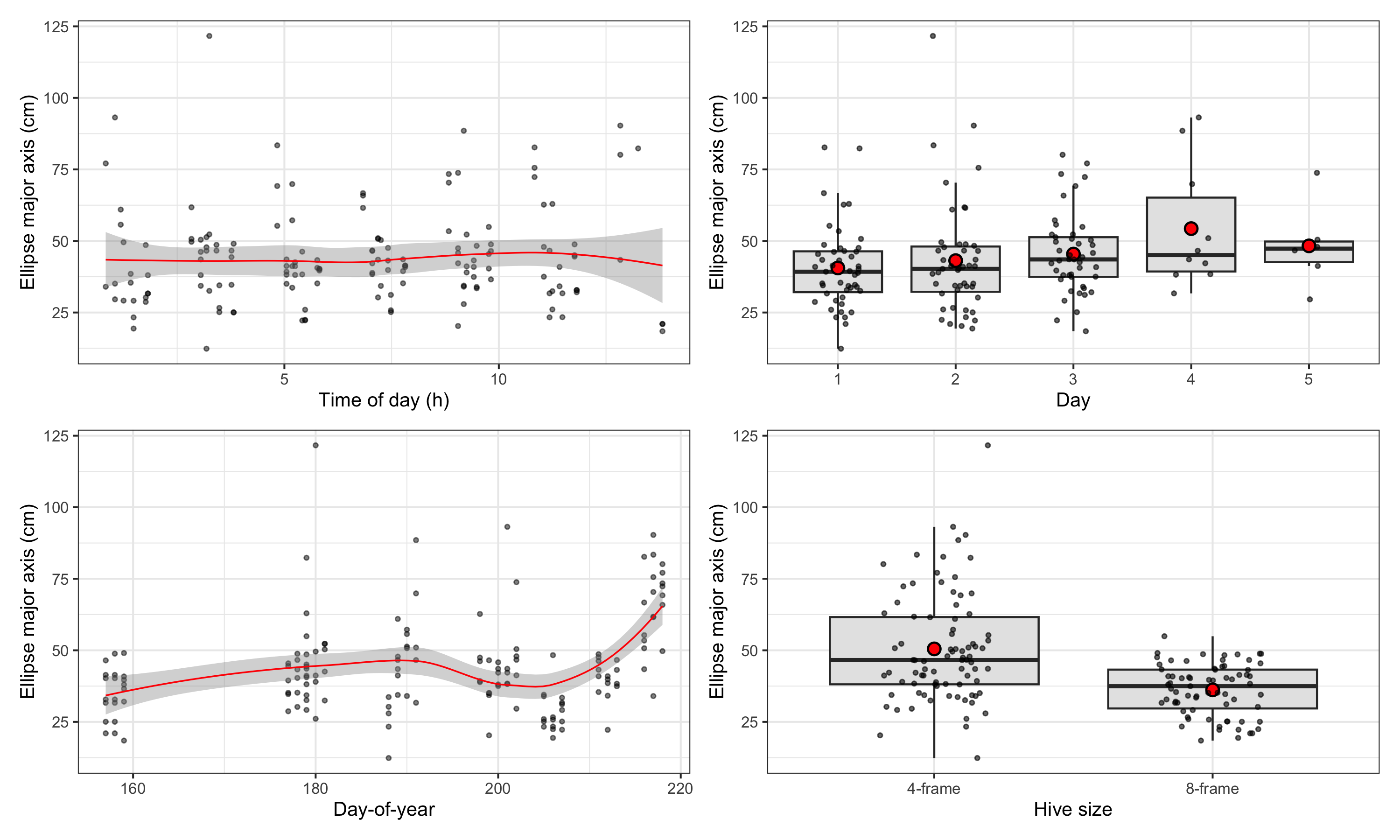

Supplement: S5 Fig — The variables TrialDay (P = 0.0062) and HiveSize (P = 0.0413) significantly predicted dance floor length. Plot elements are as described in S1 Fig. (PNG) [file pone.0341456.s005.png]

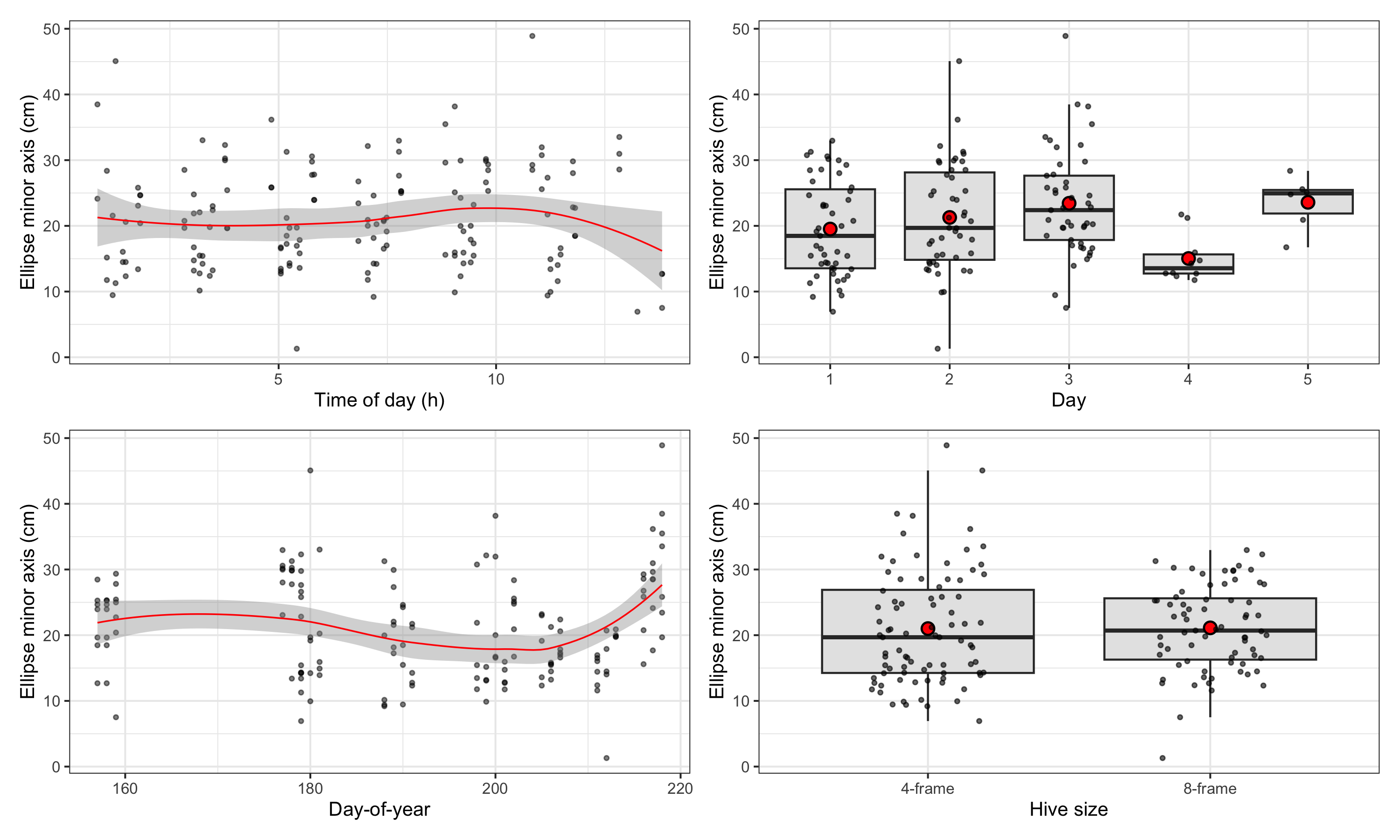

Supplement: S6 Fig — Hive size significantly predicted dance floor width (P = 0.0033). Plot elements are as described in S1 Fig. (PNG) [file pone.0341456.s006.png]

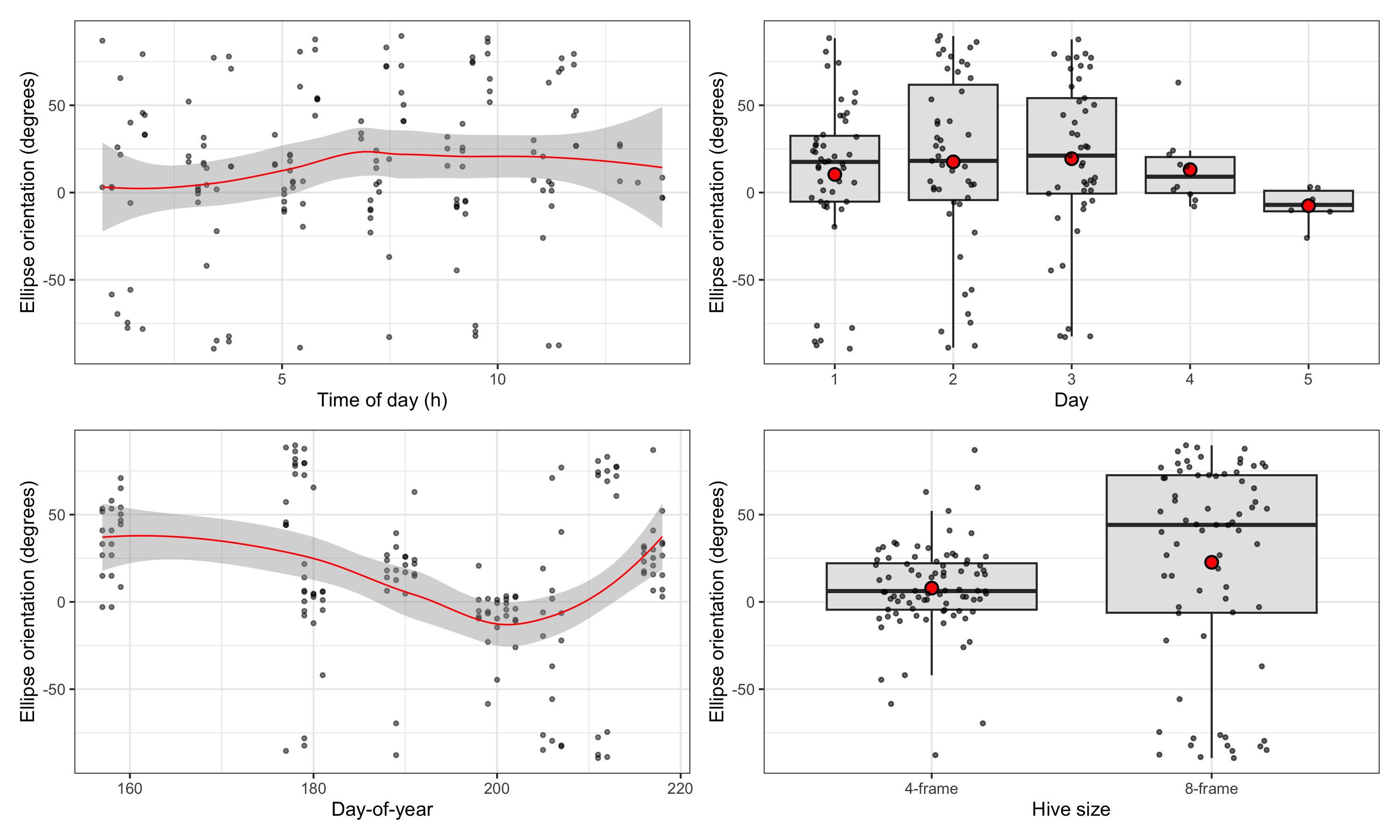

Supplement: S7 Fig — No variables had a main effect on the angle of the major axis of the dance floor. Plot elements are as described in S1 Fig. (PNG) [file pone.0341456.s007.png]

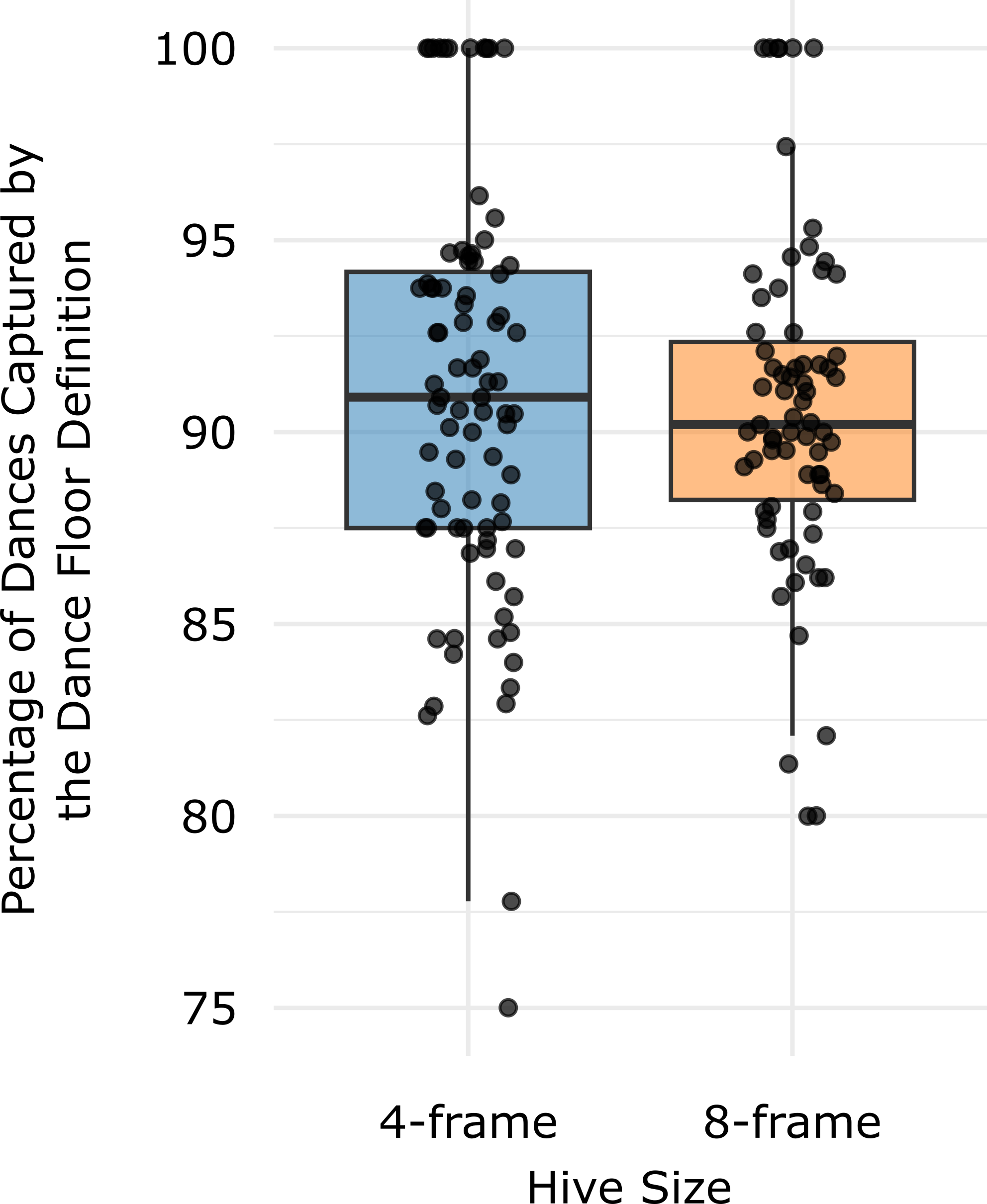

Supplement: S8 Fig — Across all colonies, the dance floor definition consistently captured approximately 91% of waggle dances. There was no significant difference in capture rate between 4-frame and 8-frame hives. (PNG) [file pone.0341456.s008.png]
