# Supplementary material for: Quantifying the honey bee dance floor: A data-driven method for defining and comparing waggle dance regions
Source: PLoS One. 2026 Feb 18;21(2):e0341456. doi: 10.1371/journal.pone.0341456 (PMC12915931; doi:10.1371/journal.pone.0341456)
Supplement: S1 Table — (DOCX) [file pone.0341456.s009.docx]

**S1 Table. Metrics of dance floors illustrated in Figs 1–4.**

| Figure | Observation | Area (cm^2^) | Perimeter (cm) | Centroid X (cm) | Centroid Y (cm) | Length (cm) | Width (cm) | Angle  (°) | Total dances | Captured dances |
| --- | --- | --- | --- | --- | --- | --- | --- | --- | --- | --- |
| 1B | Trial 1, Day 1, 1400–1430 | 648.4 | 96.3 | 20.6 | 19.8 | 41.4 | 25.3 | 40.9 | 105 | 96 (91.4%) |
| 2A | Trial 2, Day 1, 1000–1030 | 667.1 | 95.5 | 19.9 | 20.2 | 34.7 | 30.0 | –85.6 | 98 | 83 (84.7%) |
| 2A | Trial 2, Day 1, 1400–1430 | 826.6 | 104.9 | 23.1 | 21.2 | 39.5 | 33.0 | 57.2 | 84 | 77 (91.7%) |
| 2A | Trial 2, Day 1, 1800–1830 | 519.9 | 92.7 | 24.6 | 20.8 | 44.5 | 28.0 | 44.1 | 12 | 11 (91.7%) |
| 2B | Trial 5, Day 1, 0930–1000 | 240.1 | 63.6 | 16.4 | 13.4 | 32.6 | 15.4 | 14.0 | 16 | 14 (87.5%) |
| 2B | Trial 5, Day 2, 1330–1400 | 346.1 | 76.9 | 20.7 | 14.1 | 34.3 | 14.2 | 0.3 | 76 | 72 (94.7%) |
| 2B | Trial 5, Day2, 1730–1800 | 468.1 | 110.5 | 26.4 | 12.9 | 62.9 | 13.4 | –7.8 | 43 | 39 (90.7%) |
| 4A | All 8-frame trials, days, times | 842.4 | 105.7 | 20.2 | 20.5 | 40.3 | 28.0 | 75.9 | 4,419 | 3,881 (87.8%) |
| 4B | All 4-frame trials, days, times | 1,155.1 | 129.2 | 26.2 | 19.1 | 56.0 | 28.9 | 19.6 | 2,997 | 2,612 (87.2%) |
